# Loss-of-Function Variants in *TBC1D32* Underlie Syndromic Hypopituitarism

**DOI:** 10.1210/clinem/dgaa078

**Published:** 2020-02-15

**Authors:** Johanna Hietamäki, Louise C Gregory, Sandy Ayoub, Anna-Pauliina Iivonen, Kirsi Vaaralahti, Xiaonan Liu, Nina Brandstack, Andrew J Buckton, Tiina Laine, Johanna Känsäkoski, Matti Hero, Päivi J Miettinen, Markku Varjosalo, Emma Wakeling, Mehul T Dattani, Taneli Raivio

**Affiliations:** 1 Pediatric Research Center, Helsinki University Hospital, New Children’s Hospital, Pediatric Research Center, Helsinki, Finland; 2 Genetics and Genomic Medicine Programme, UCL Great Ormond Street Institute of Child Health, London, UK; 3 North West Thames Regional Genetic Service, London North West University Healthcare NHS Trust, Harrow, UK; 4 Department of Physiology, Medicum Unit, and Translational Stem Cell Biology and Metabolism Research Program, Faculty of Medicine, University of Helsinki, Helsinki, Finland; 5 Institute of Biotechnology & HiLIFE, University of Helsinki, Helsinki, Finland; 6 Department of Radiology, Helsinki University Hospital and University of Helsinki, Helsinki, Finland; 7 London North Genomic Laboratory Hub, Great Ormond Street Hospital NHS Trust, London, UK; 8 Molecular Basis of Rare Diseases Section, Genetics and Genomic Medicine Programme, UCL Great Ormond Street Institute of Child Health, London, UK; 9 Department of Endocrinology, Great Ormond Street Hospital for Children, London, UK

**Keywords:** TBC1D32, hypopituitarism, Sonic Hedgehog signaling, ciliopathy, retinal dystrophy

## Abstract

**Context:**

Congenital pituitary hormone deficiencies with syndromic phenotypes and/or familial occurrence suggest genetic hypopituitarism; however, in many such patients the underlying molecular basis of the disease remains unknown.

**Objective:**

To describe patients with syndromic hypopituitarism due to biallelic loss-of-function variants in *TBC1D32*, a gene implicated in Sonic Hedgehog (Shh) signaling.

**Setting:**

Referral center.

**Patients:**

A Finnish family of 2 siblings with panhypopituitarism, absent anterior pituitary, and mild craniofacial dysmorphism, and a Pakistani family with a proband with growth hormone deficiency, anterior pituitary hypoplasia, and developmental delay.

**Interventions:**

The patients were investigated by whole genome sequencing. Expression profiling of *TBC1D32* in human fetal brain was performed through in situ hybridization. Stable and dynamic protein-protein interaction partners of TBC1D32 were investigated in HEK cells followed by mass spectrometry analyses.

**Main Outcome Measures:**

Genetic and phenotypic features of patients with biallelic loss-of-function mutations in *TBC1D32.*

**Results:**

The Finnish patients harboured compound heterozygous loss-of-function variants (c.1165_1166dup p.(Gln390Phefs*32) and c.2151del p.(Lys717Asnfs*29)) in *TBC1D32*; the Pakistani proband carried a known pathogenic homozygous *TBC1D32* splice-site variant c.1372 + 1G > A p.(Arg411_Gly458del), as did a fetus with a cleft lip and partial intestinal malrotation from a terminated pregnancy within the same pedigree. *TBC1D32* was expressed in the developing hypothalamus, Rathke’s pouch, and areas of the hindbrain. *TBC1D32* interacted with proteins implicated in cilium assembly, Shh signaling, and brain development.

**Conclusions:**

Biallelic *TBC1D32* variants underlie syndromic hypopituitarism, and the underlying mechanism may be via disrupted Shh signaling.

Pituitary hormone deficiencies in combination with extrapituitary manifestations suggest a role for genes involved in the early patterning of the pituitary, such as *HESX1*, *PITX2*, *OTX2*, *SOX2*, *SOX3*, *LHX3*, *LHX4*, *GLI2*, and *FGF8* that form a complex cascade culminating in the formation of midline anterior brain and craniofacial structures (1–6). However, in the majority of cases, the underlying molecular basis remains unknown. The Sonic Hedgehog (Shh) signaling pathway is essential for central nervous system (CNS), early pituitary and ventral forebrain development in mice. Pathogenic variants in components of the SHH pathway have been described in patients with holoprosencephaly, isolated congenital hypopituitarism, and cranial/midline facial abnormalities (7). Variably penetrant variants in *GLI2*, a zinc-finger transcription factor that mediates SHH transduction, have been described in patients with variable holoprosencephaly phenotypes, including microcephaly, bilateral cleft lip/palate, postaxial polydactyly, optic nerve hypoplasia, and an absent/hypoplastic pituitary with isolated or multiple anterior pituitary hormone deficiencies (8). *GLI2* variants are not infrequently associated with combined pituitary hormone deficiency in isolation, without midline defects or other features of holoprosencephaly (9).

Extrapituitary phenotypic features in patients with complex forms of hypopituitarism may be suggestive of certain genotypes, and are important in guiding molecular studies (1). Herein, we utilized whole genome sequencing (WGS) to identify the molecular basis of hypopituitarism in 3 patients who presented with pituitary hormone deficiencies and craniofacial phenotypes, together with variable limb, intellectual, and retinal phenotypes. Our results show that the patients carried biallelic loss-of-function variants in *TBC1D32*, a gene implicated in ciliary function and Sonic Hedgehog signaling (10, 11). We subsequently investigated the expression of *TBC1D32* in the developing human brain and examined its protein–protein interaction partners to gain insight into the putative disease mechanism by which variants in this gene cause such a complex phenotype.

## Materials and Methods

### Patients and clinical data

**Figure 1.**
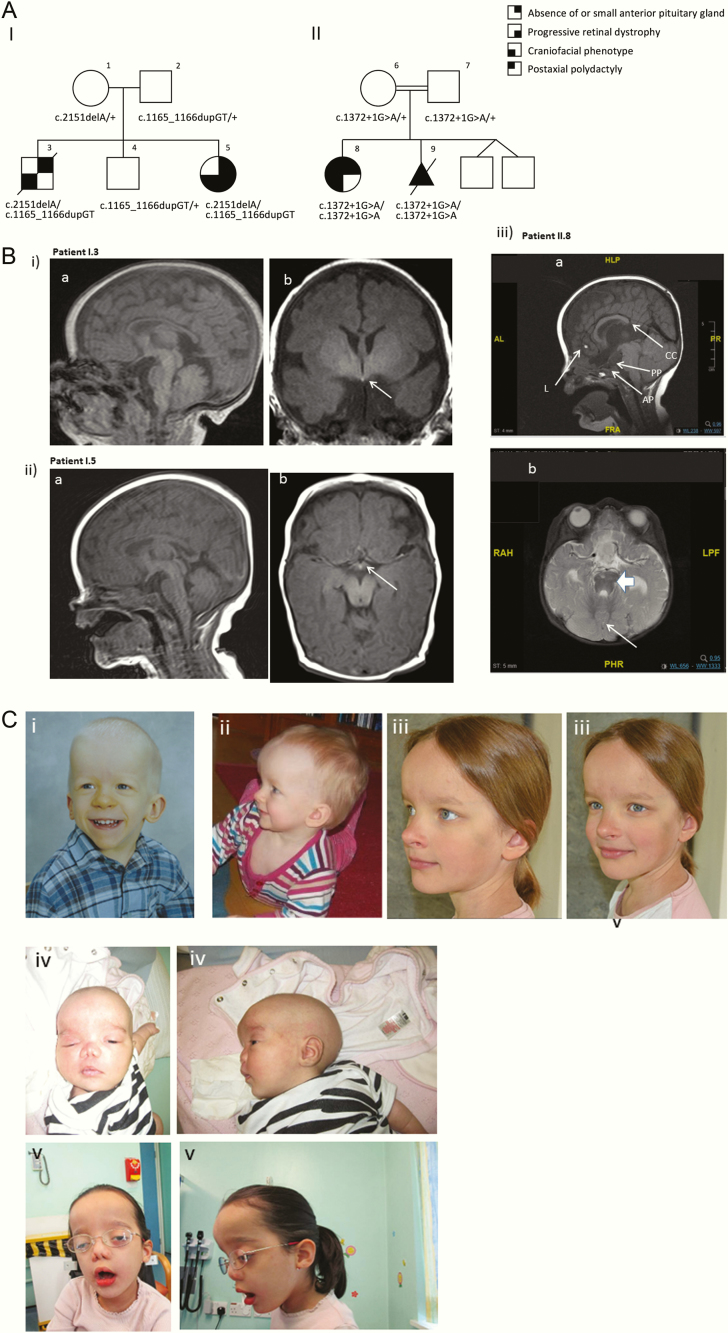
**A:** The pedigrees of our patients with hypopituitarism and biallelic *TBC1D32* variants. I: the Finnish pedigree; II: the Pakistani pedigree. Patients I.3 and I.5 carried compound heterozygous and patients II.8 and II.9 carried homozygous variants in *TBC1D32*. The parents were heterozygous carriers of the respective variants. **B:** (i) and (ii) the MRIs of the 2 Finnish patients. *Upper row,* Patient I.3: Sagittal **(a)** and coronal **(b)** T1 images without contrast enhancement. The sella turcica and the pituitary gland are not identifiable. Neurohypophyseal bright tissue is seen near the tuber cinereum (arrow). *Lower row*, Patient I.5: Sagittal **(a)** and axial **(b)** T1 images without contrast enhancement. The sella turcica and the pituitary gland are absent. Potentially neurohypophyseal bright tissue is seen near the tuber cinereum (arrow). (iii) MRI of patient II.8. *Upper row,* Patient II.8: Sagittal **(a)** T1 image showing partial agenesis of corpus callosum (CC), small interhemispheric lipoma (L), small anterior pituitary (AP), and small ectopic posterior pituitary (PP). *Lower row*: coronal image showing dysplasia of the cerebellar vermis (arrow) with an abnormal left cerebellum. The “molar tooth” sign of Joubert syndrome is also shown (filled arrow). **C:** Clinical photos of the patients. (i) Patient I.3 at 2.4 years of age. Note the prominent forehead and the low-set, posteriorly rotated ears; (ii) Patient I.5 presented with prominent forehead, large anterior fontanelle, and low-set ears in infancy; (iii) Patient I.5 at 10.5 years of age; (iv) Patient II.8 in infancy showing a prominent forehead with hypertelorism, low-set ears, flat nasal bridge and anteverted nares; (v) Patient II.8 at 5.5 years of age.

We investigated a Finnish family (Pedigree I, Fig. 1A) including 2 affected siblings with an absent anterior pituitary, ectopic posterior pituitary, mild craniofacial dysmorphism, and progressive retinal dystrophy, and a consanguineous Pakistani family (Pedigree II, Fig. 1A) with a proband who presented with developmental delay and features suggestive of oral-facial-digital syndrome (OFDS) associated with growth hormone (GH) deficiency. Detailed pedigrees are documented in the Results section.

### Genetics

Genomic DNA was extracted from the subject’s peripheral blood leukocytes. WGS of the Finnish family was performed in the Beijing Genomic Institute (BGI, Shenzhen, China) with Illumina HiSeq X Ten technology. On the basis of pedigree I, a recessive mode of inheritance was assumed to be most likely. Therefore, filtering analysis of the WGS data searched for either homozygous or compound heterozygous variants that were present in both patients, where the parents carried only 1 in a heterozygous state. We only considered non-synonymous or splice site variants that were novel or had frequencies of less than 0.5%. WGS of the Pakistani family was carried out via the 100 000 Genomes Project: Protocol v3, Genomics England. (12) A number of standardized panels from Genomics England PanelApp were subsequently applied to the data from Pedigree II (13), including a panel for rare, multisystem ciliopathy disorders (v1.28). Targeted sequence analysis by bidirectional Sanger sequencing was used to confirm the presence and the mono-/biallelicity of the likely pathogenic variants.

### Reverse transcriptase polymerase chain reaction analysis

A 310-bp fragment of the *TBC1D32* transcript was amplified from the human pituitary gland and hypothalamic cDNA, respectively, (QUICK-Clone pituitary cDNA, Takarabio, 1.5µl / reaction; Hypothalamus Marathon®-Ready cDNA, Takarabio, 1.5ul / reaction) using cDNA-specific primers. *GAPDH* served as a reference gene. The polymerase chain reaction (PCR) products were visualized on a 1% agarose gel.

### Human embryonic expression analysis: in situ hybridisation

A purified pT7T3D-PacI vector containing a portion of the human wild-type *TBC1D32* cDNA (IMAGE ID: 505 804) (Source Bioscience) was used to make both the antisense and control sense digoxigenin-labeled *TBC1D32* RNA probes. Human embryonic tissue sections were selected at Carnegie stage (CS) 19, 20, and 23 (equivalent to gestational age 6, 7, and 8 weeks into development). respectively, obtained from the Human Developmental Biology Resource (HDBR). Due to limited access to human embryonic tissue, we were restricted to these 3 stages during embryogenesis. The in situ hybridisation protocol was performed as previously described (14) to generate a human embryonic expression brain profile incorporating the hypothalamo-pituitary region. More detailed information on restriction enzymes and RNA sequences are available upon request.

### Cell culture, affinity purification, and mass spectrometry

The *TBC1D32* PCR product with flanking at B sites was used for BP reaction to generate the gateway compatible entry clone. LR recombination was performed between the entry clones and the destination vector to generate the MAC-tag tagged TBC1D32 expression vector (15). Culture of Flp-In T-REx 293 (ThermoFischer Scientific, Waltham, Massachusettes) cell lines, transfection, and stable cell line selection were performed as previously described (16, 17). Affinity purification (AP) and BioID experiments, together with mass spectrometry (MS) analysis, were performed as previously described (18).

### Ethics

The study was approved by the Ethics Committee of the Hospital District of Helsinki and Uusimaa (Committee for women, children and psychiatry, approval HUS/3325/2017). Full informed consent was obtained from the United Kingdom family to participate in the 100 000 Genomes Project. The guardians of the patients gave their written informed consent to participate in the phenotyping and genetic studies. Written consent for publication of photographs was also obtained from the parents of the proband in the Pakistani family and from the parents of the Finnish family.

## Results

### Pedigree I

A Finnish family with healthy parents, 2 affected children and 1 unaffected child, is shown in Fig. 1A. Patient I.3 (Fig. 1A) was born at 42 + 1 weeks gestation following an uncomplicated pregnancy. Labor was induced due to postmaturity and proceeded to an emergency caesarean section due to changes in the cardiotocography (CTG)-scan. The patient had birth asphyxia with Apgar scores of 1/4/5 at 0/5/15 minutes of age, respectively. He was hydropic, hypotonic, and had recurrent hypoglycemia, jaundice, and transient diabetes insipidus. Although the post-term ultrasound scan at gestational week (GW) 41 + 1 had shown short fetal femoral bones, his birth length (50.2 cm, -1.0 SDS), weight (3.51 kg, -0.8 SDS), and head circumference (38.0 cm, +1.6 SDS) were within normal limits. He was treated with intermittent continuous positive airway pressure (nasal-CPAP), short intubation and phototherapy, and had a patent ductus arteriosus (PDA), which closed spontaneously, with no other structural cardiac anomalies. He had micropenis and bilateral cryptorchidism (corrected surgically before 2 years of age). During the first weeks of life, he was diagnosed with growth hormone (GH), adrenocorticotropin (ACTH), thyroid-stimulating hormone (TSH), and gonadotropin deficiencies (Table 1). Treatment with L-thyroxin, hydrocortisone, and GH was commenced immediately. Brain magnetic resonance imaging (MRI) revealed that the sella turcica and the hypophysis were not identifiable, and an ectopic or undescended neurohypophysis was present near the tuber cinereum (Fig. 1B). The optic chiasm was somewhat narrow, although the optic nerves were normal. In addition to the endocrine abnormalities, he had communicating hydrocephalus, developmental delay, and slight bilateral astigmatism. He was partially dependent on nasogastric tube feeding up to 5 months of age and had severe secretory otitis media with insertion of grommets at the age of 1 year. At 3 years of age, he died unexpectedly following infection, in spite of adequate hydrocortisone substitution.

**Table 1.** Biochemical testing of the pituitary hormone secretion in Patients I.3 and I.5

	Patient I.3		Patient I.5	
	**Age (Days)**	**Test Result [NR]**	**Age (Days)**	**Test Result [NR]**
**Growth hormone**				
Serum IGF1 (nmol/L)	8	7 [na]	20	<3 [7–43]
Fasting serum GH during hypoglycemia (ug/L)	8	<0.03	6	0.20
Maximum arginine-stimulated serum GH (ug/L)	-	NA	26	0.23
**ACTH**				
Plasma ACTH (ng/L)	14	<5^a^ [10–50]	11	15^a^ [<46]
Serum cortisol (nmol/L)	1	<20 [150–650]	11	121^a^ [30–632]
Maximum synacthen-stimulated plasma cortisol (ACTH neo test) (nmol/L)	1	<20	11	638^a^
**TSH**				
Serum free T4 (pmol/L)	4	5.3 [9–19]	20	9.9 [8–25]
Serum TSH (mU/L)	4	0.002 [0.6–10]	20	2.65 [0.6–10]
Serum TSH during TRH provocation test (at 0/20/60 min) (mU/L)		NA	26	2.58/4.63/4.73
**Gonadotropins**				
Serum inhibin B (ng/L)	2	175	-	NA
Serum FSH (IU/L)	18	<0.10	-	NA
Maximum serum FSH during GnRH provocation test (IU/L)	18	0.2	-	NA
Serum LH (IU/L)	18	<0.10	-	NA
Maximum serum LH during GnRH provocation test (IU/L)	18	<0.10	-	NA

Abbreviations: NA, not available; NR, normal range.

^a^Measured during exogenous cortisone treatment.

Patient I.5 (Fig. 1A), the sister of I.3, was born by vaginal delivery at 41 + 6 weeks gestation following an uncomplicated pregnancy. Her birth length, weight, and head circumference were 51 cm (-0.1 SDS), 3.90 kg (+0.1 SDS), and 37 cm (+1.3 SDS), respectively. She had a prominent forehead, large anterior fontanelle, and deep set eyes, with low-set ears (Fig. 1C). She had hypotonia, hypoglycemia, and metabolic acidosis. Endocrine investigations were initiated soon after birth due to her phenotype and a positive family history of hypopituitarism. She was diagnosed with GH and TSH deficiencies (Table 1) and has received GH and L-thyroxin substitution treatment since the neonatal period. In addition, the patient was commenced on oral hydrocortisone due to the family history and the possibility of progressive ACTH deficiency. Magnetic resonance imaging revealed an absent sella turcica and anterior pituitary gland, and potential neurohypophyseal tissue was detected close to the tuber cinereum (Fig. 1B). She had insertion of grommets before 1 year of age. Cardiac ultrasound and 24-hour electrocardiogram (ECG) were both normal at the age of 6 months. Interestingly, she has also been diagnosed with progressive retinal dystrophy, motor delay, neuromuscular scoliosis, and discrepancy of her lower limb length. Her upper jaw is narrow with misaligned teeth, of which 3 have by now been extracted. Due to marked feeding difficulties, she underwent speech therapy until 6 years of age. At the age of 8 years, she was prepubertal and exhibited dysmorphic craniofacial features, including a prominent forehead, wide nasal bridge, short upturned nose, hypertelorism, downward slanting palpebral fissures, posteriorly-rotated ears, low hairline, and nuchal hair (Fig. 1C). She had a barrel-like chest and widely spaced nipples. Hypermobility was noted in the upper limbs. Her finger pads were prominent and she had fingernail clubbing. There was slight syndactyly of the 2nd and 3rd toes, and a bilateral sandal gap. Her cognitive development is normal.

Whole genome sequencing data had an average sequencing depth of at least 28.83 and 99.01% coverage for each sample. Both I.3 and I.5 carried compound heterozygous variants predicted to lead to frameshifts and premature stop codons, c.1165_1166dup p.(Gln390Phefs*32) and c.2151del p.(Lys717Asnfs*29), in the *TBC1D32* (NM_152730.5) gene (Fig. 1A). The mother (I.1) carried the c.2151del variant, and the father (I.2) and healthy brother (I.4) carried the c.1165_1166dup variant in a heterozygous state. The c.1165_1166dup p.(Gln390Phefs*32) (rs546631812) variant is reported in the gnomAD database with a minor allele frequency of 0.001090. The c.2151del p.(Lys717Asnfs*29) variant is absent from the gnomAD, ExAC, and SISu Project databases.

### Pedigree II

An independent consanguineous pedigree with a loss-of-function variant in *TBC1D32* is presented in Fig. 1A. The proband (II.8) is a 5-year-old girl, born to second cousin Pakistani parents (II.6 and II.7). Antenatal scans showed short long bones (femur length below 3^rd^ percentile), midline cystic changes in the brain, and polyhydramnios. Her birth weight at term was 3.27 kg (9^th^ percentile; -0.4 SDS). She was noted to have distinctive facial features with a broad forehead, wide anterior fontanelle, hypertelorism, low-set ears, flat nasal bridge, anteverted nares, slight midline groove on the tongue, serrated gums (but not overt frenulae), ankyloglossia, and a high, narrow palate (Fig. 1C). Other findings included postaxial polydactyly on her left hand, small hands, and apparent rhizomelic shortening of the limbs. After failure to pass a nasogastric tube she was found to have left-sided choanal atresia. An MRI brain scan performed at birth was reported as showing partial agenesis of the corpus callosum and a very small anterior pituitary gland and optic chiasm, consistent with septo-optic dysplasia (Fig. 1B). There was dysplasia of the cerebellar vermis with a midline cleft, similar to MRI scan findings observed in Joubert syndrome. Her brainstem was small, with a small interhemispheric lipoma. A skeletal survey, renal ultrasound and echocardiogram were all normal. Her head circumference at 6 months was 42.5 cm (+0.1 SDS). Her growth was suboptimal with a GH concentration of 0.6 ug/L in response to hypoglycaemia with an undetectable serum IGF-1 and a low IGFBP3 (0.75 mg/L; NR 0.8–3.9). Her cortisol was normal at 464 nmol/L. She was commenced on GH treatment at 13 months of age (height SDS -5.0) with a good response. At 4.75 years, her height was 91.2cm (-3.2 SDS).

She has global developmental delay and attends a special needs school. She sat independently at 2 years. However, at 5.5 years she is still unable to mobilize independently, although she will stand with support. She does not vocalize but understands familiar words. At 3 years of age, she had grommet insertion for bilateral glue ear and her hearing is currently normal. She has a bilateral divergent squint and severe cerebral visual impairment with reduced visual acuity but with normal retinal responses on electroretinogram. Whole genome sequencing showed that patient II.8 was homozygous for a variant (NM_152730.5; c.1372 + 1G > A) in *TBC1D32* (Fig. 1A). This variant was previously reported in association with OFDS type IX (19) and is predicted to cause aberrant splicing by abolishing the exon 12 splice site, resulting in exon skipping of exon 12, with a truncation of the protein, p.(Arg411_Gly458del), and was therefore considered pathogenic. In her mother’s second pregnancy, the fetus (II.9) was found to have midline facial clefting and a femoral length below the 3rd percentile on ultrasound scan at 20 weeks gestation. The parents opted for a termination of the pregnancy. Postmortem examination showed a female fetus with midline cleft lip and partial intestinal malrotation. Targeted sequence analysis by bidirectional Sanger sequencing confirmed the presence of the homozygous variant in both the proband and fetus (Fig. 1A). Parental testing, also by Sanger sequencing, confirmed their carrier status. Subsequently, the mother has given birth to healthy twin boys, who have not been genetically tested.

### Human embryonic and adult TBC1D32 expression analysis

**Figure 2.**
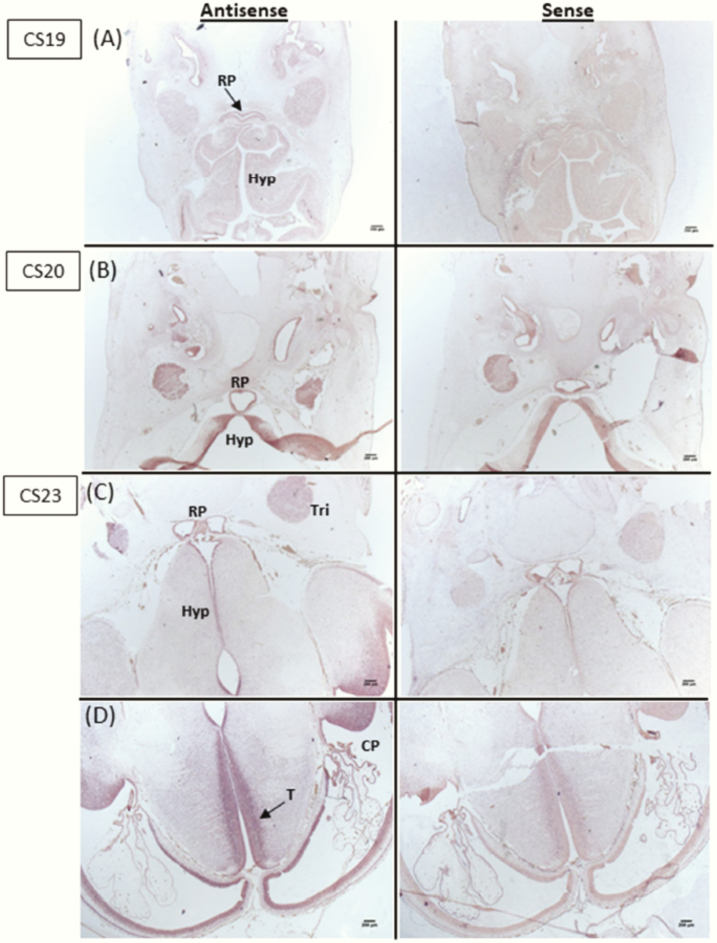
Human expression of *TBC1D32* mRNA transcripts in transverse brain sections at different developmental stages during embryogenesis. **A:** There is no clear expression in the hypothalamus, Rathke’s pouch, or elsewhere in the brain when comparing results using the antisense probe and the sense probe at Carnegie stage (CS) 19. **B:** At CS20 there may be some partial expression in the hypothalamus using the antisense probe; however, staining is very similar to the Rathke’s pouch and the hypothalamus when using the control sense probe, where some background staining is noted. **C:** At CS23, there is partial expression in the trigeminal ganglia, in Rathke’s pouch, and along the hypothalamus when comparing the antisense and control sense probes. **D:** There is strong expression in the hindbrain, in particular the thalamus, with some expression also seen in the choroid plexus. Abbreviations: CP, choroid plexus; Hyp, hypothalamus; RP, Rathke’s pouch; T, thalamus; Tri, trigeminal ganglia.

**Figure 3.**
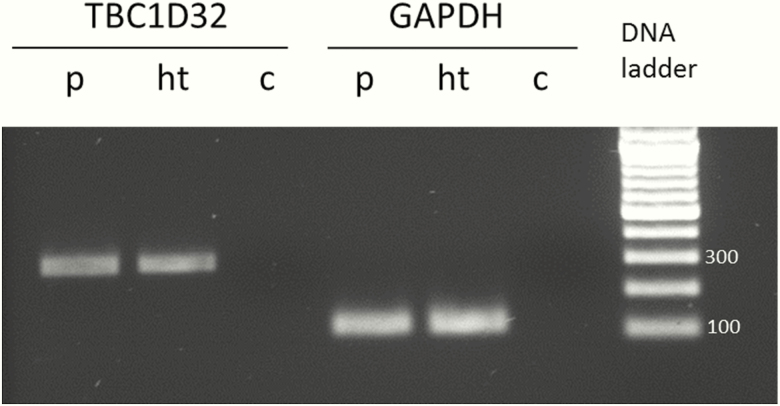
Reverse transcriptase PCR analysis of *TBC1D32* expression. A 310-bp fragment of transcript encoding *TBC1D32* was amplified from human pituitary gland cDNA and from hypothalamic cDNA (QUICK-Clone pituitary cDNA, Takarabio, 1.5µl / reaction; Hypothalamus Marathon®-Ready cDNA, Takarabio, 1.5ul / reaction). Human *GAPDH* was used as a reference gene. The PCR products were visualized on a 1.0% agarose gel. Abbreviations: c, negative control without DNA template; ht, hypothalamus; p, pituitary gland.

In situ hybridization studies showed that human *TBC1D32* expression during embryonic brain development is not prominent in the hypothalamus or Rathke’s pouch at Carnegie stage (CS) 19 or 20 (Fig. 2A–B). However, at CS23, *TBC1D32* is expressed in the hypothalamus, Rathke’s pouch, trigeminal ganglia, and choroid plexus (Fig. 2C,D), with strong expression throughout the hindbrain and thalamus at this stage (Fig. 2D). Additionally, *TBC1D32* is expressed in both adult human pituitary and hypothalamic cDNA libraries (Fig. 3).

### Protein–protein interaction partners of TBC1D32

**Figure 4.**
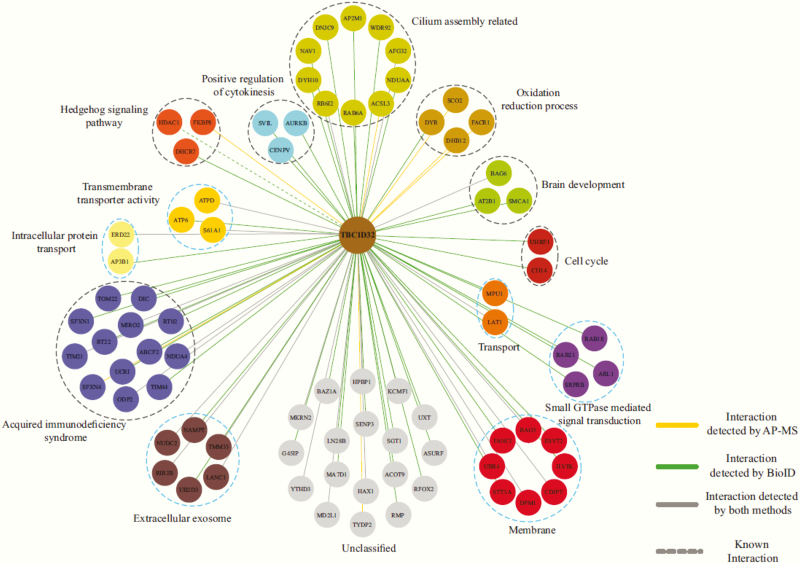
The functional grouping of the high confidence interacting proteins of TBC1D32. Interactome analysis reveals known and novel interactions for TBC1D32. AP-MS and BioID analysis of TBC1D32 identified 81 high-confidence protein–protein interactions (yellow lines represent interaction detected by AP-MS approach; green lines represent interactions detected by BioID approach; overlap of the 2 purification methods is shown with grey lines; known interaction is shown with a dashed line). The interacting proteins are grouped based on their molecular functions/complexes.

We employed a MAC-tag approach (15) to comprehensively identify the interaction partners of TBC1D32 (a.k.a broad-minded, BROMI) by performing affinity purification (AP) and proximity labeling (BioID), followed by MS. Using a comparative statistical analysis (15), we identified a total of 81 high confidence interacting proteins (HCIPs) (20), and subsequently, the DAVID functional annotation clustering tool (21) and primary cilium database (22) were used to group the related proteins. The results are shown in Fig. 4. In brief, the 81 interactors were mapped to 13 different cellular gene ontology classes: hedgehog signaling, cilium assembly, extracellular exosome, membrane, small GTPase-mediated signal transduction, transport, cell cycle, brain development, oxidation reduction process, positive regulation of cytokinesis, transmembrane transporter activity, acquired immunodeficiency syndrome, and intracellular protein transport. The analysis disclosed 25 novel interactors of TBC1D32 that were grouped in the following six classes: transport; small GTPase mediated signal transduction; membrane; intracellular protein transport; transmembrane transporter activity; extracellular exosome (Fig. 4). We performed both AP and proximity labeling (BioID) coupled with MS for TBC1D32. The TBC proteins are a group of Rab-GAP (GTPase-activating protein) proteins, which are involved in the plasma membrane-endosome trafficking processes (23). Another critical group of proteins interacting with TBC1D32 (13 prey proteins) belongs to acquired immune deficiency syndrome and provides a putative link between the immune system and TBC1D32 function. Notably, 4 interacting proteins were associated with the oxidation-reduction process, and 3 proteins were linked to brain development.

## Discussion

To date, biallelic *TBC1D32* variants have been described in 1 male patient with severe facial and ocular phenotypes, microcephaly, postaxial polydactyly, and CNS abnormalities, including the absence of the pituitary gland with subsequent panhypopituitarism (19). Herein, we describe phenotypic features of 3 children and 1 aborted fetus from 2 unrelated pedigrees that are affected by biallelic loss-of-function variants in *TBC1D32.* Our results, together with those of Adly et al, confirm that *TBC1D32* variants underlie a recessive disorder demarcated by severe but highly variable expression of midline defects, including congenital hypopituitarism due to abnormal hypothalamo-pituitary development.

Oral-facial-digital syndromes are clinically and genetically heterogeneous ciliopathies characterized by abnormalities of the face, oral cavity, and extremities (24). To date, at least 13 OFDS subtypes and 16 associated genes have been reported (24, 25). Comparison of the phenotypic features of the patients in our study and the patient with OFDS type IX reported by Adly et al (19) is shown in Table 2. It is noteworthy that the recurring splice-site variant is associated with postaxial polydactyly, but variable ocular (reduced visual acquity or microphthalmia with coloboma) and oral (highly arched/ cleft palate with or without midline tongue groove) phenotypes (Table 2), consistent with variable expressivity. In contrast, the Finnish siblings carrying 2 other loss-of-function variants in *TBC1D32* did not exhibit postaxial polydactyly but had variable intellectual and ocular phenotypes. A unifying theme in all 4 patients reported to date, however, is the pituitary gland phenotype, with variable pituitary hormone deficiencies (Table 2). The CNS phenotype of the aborted fetus is not available; however, the fetus also carried the homozygous *TBC1D32* p.Arg411_Gly458del truncation and displayed cleft lip and intestinal malrotation, adding the latter feature to the list of associated phenotypes in biallelic *TBC1D32* loss-of-function variant carriers. Human gene expression analysis in our study identified *TBC1D32* expression in the developing hypothalamus and pituitary gland (CS23) (Fig. 2C) and in the pituitary and hypothalamic cDNA libraries of adults (Fig. 3), suggesting activation of this gene during the early stages of hypothalamo-pituitary formation during embryogenesis, which is maintained into adulthood. Furthermore, the strong *TBC1D32* expression seen in other areas of the hindbrain (Fig. 2D) suggests that *TBC1D32* plays a role in other regions of the brain during development, possibly reflective of the complex variable CNS problems seen in OFDS patients such as ours.

**Table 2.** Summary of clinical features in patients with biallelic mutations in *TBC1D32*

	Subject I.3	Subject I.5	Subject II.8	Subject II.9	Adly et al (2014) (19)
**Age**	3 years^*a*^	10 years	5 years	20 weeks gestation	6 months^*a*^
**Sex**	Male	Female	Female	Female	Male
**Oral anomalies**					
Bifid tongue/midline tongue groove	-	-	+	-	-
Highly arched/cleft palate	-	-	+	-	+
Abnormal dentition	-	+	-	NA	-
**Facial anomalies**					
Hypertelorism	+	+	+	-	+
Midline cleft lip	-	-	-	+	+
Upturned nose	+	+	+	NA	NA
Choanal stenosis/atresia	-	-	+	NA	+
**Limb anomalies**					
Polydactyly	-	-	+	-	+
Syndactyly	-	+	-	-	-
Sandal gap deformity	-	+	-	-	NA
Lower limb length difference	NA	+	-	NA	NA
**CNS anomalies**					
Cerebellar vermis hypoplasia	-	-	+	NA	+
Hypothalamic hamartoma	-	-	-	NA	-
Agenesis of the corpus callosum	-	-	+	NA	+
Pituitary abnormalities	No anterior pituitary (ACTH, TSH, GH, and FSH/LH deficiencies), ectopic posterior pituitary	No anterior pituitary (GH, TSH deficiencies), ectopic posterior pituitary	Anterior pituitary hypoplasia (GH deficiency)	NA	No pituitary gland
Basal ganglia abnormalities	-	-	+	NA	-
Brainstem abnormalities	-	-	+	NA	-
Communicating hydrocephalus	+	-	-	NA	-
**Eye involvement**					
Microphtalmia	-	-	-	NA	+
Retinal dystrophy	NA	Progressive	-	NA	-
Coloboma	-	-	-	NA	+
Visual acuity	NA	Reduced	Reduced	NA	NA
**Other**					
Microcephaly	-	-	-	-	+
Developmental delay	Global	Motoric	Global	NA	NA
Epileptic seizures	-	-	-	NA	+
Congenital heart disease	-	-	-	-	+
Abnormal genitalia	+	-	-	-	+
Intestinal malrotation	-	-	-	+	-
Neuromuscular scoliosis	NA	+	-	NA	NA
Chronic secretory otitis media	+	+	+	NA	NA

Abbreviations: +, present; -, not present; NA, not available.

^*a*^Age at death.

Previous proteomic analysis of cilia has confirmed that TBC1D32 is a ciliary protein (26). Cilia can be motile or nonmotile (primary cilia) and are specialized microtubule-based sensory organelles (27), which play a role in cell polarity determination and in mediating vital signaling cascades such as the SHH signaling pathway (28). Therefore, the correct development of cilia is a prerequisite for SHH signaling. Deletion of the *Tbc1d32* homolog in mice, referred to as the *Bromi* mouse, causes a severe phenotype encompassing developmental defects, including a lack of the neural tube floor plate, exencephaly, signs of defective ventral neural fate specification, poorly developed eyes, and preaxial polydactyly. Electron microscopy of the neuroepithelium in these mice showed curled axonemes surrounded by dilated ciliary membranes. These studies showed that *Bromi*-mutant neurons were associated with deficient Shh signaling activation of downstream regulators Gli2 and Gli3, and that Tbc1d32 is critical for the correct localization of Gli2 within the cilium (10). A similar phenotype to the *Bromi* mouse was also noted in morpholino-mediated knockdown studies of the gene homolog in zebrafish (10). Hence, these studies provide a clear link between ciliary morphology and the correct localization of Gli2 in the cilia, and suggest that the hypopituitary phenotype observed in association with *TBC1D32* variants in humans is likely the result of GLI2 mislocalization. Recently, mutation in *IFT172* has been associated with a phenotype characterized by anterior pituitary hypoplasia, an ectopic posterior pituitary, retinopathy, metaphyseal dysplasia, and renal failure (29). *IFT172* has been implicated in ciliary function, and hence our study provides further support for the role of cilia in hypothalamo-pituitary disease.

In mammalian cells, primary cilia are essential for SHH signaling (30), and in protein–protein interaction studies we identified 10 proteins of HCIPs that are involved in the assembly of primary cilia (Fig. 4). Our analyses confirmed that TBC1D32 interacts with histone deacetylase 1 (HDAC1), a protein implicated in SHH signaling (31), and interacts with 2 additional prey proteins (FKBP8, DHCR7) that regulate SHH signaling (32–37) (Fig. 4). Furthermore, the loss of mouse cell cycle-related kinase (CCRK) function, a protein known to interact with TBC1D32 (10), has been shown to result in a highly similar embryonic phenotype to that of the *Bromi*-null mouse, resulting in disrupted optic cup and lens formation, shortened optic stalk, reduced neural retina specification, and ectopic retinal pigment epithelium formation (10, 38, 39). Although our experiment using a human cell line did not find an association with CCRK, 5 TBC1D32-interacting proteins (CI114, UHRF1, AURKB, CENPV, SVIL) were associated with positive regulation of cytokinesis and the cell cycle (Fig. 4). Taken together, proteomics provided a conceivable link between TBC1D32 and the developmental defects of the pituitary gland and the eye. Although we found a connection between TBC1D32 and regulators of the immune system, its association with the recurrent ear infections seen in all the patients and the unexpected death of patient I.3 remains to be established. It is important to note, however, that patients I.5 and II.8 do not have a history of recurrent or severe infections. Immune function or ciliary function have not been examined in detail in any of the patients.

To conclude, we describe 3 patients and an aborted fetus with biallelic variants in *TBC1D32¸* a ciliary gene implicated in SHH signaling and leading to the correct localization of Gli2, a protein that is significantly implicated in the aetiology of congenital hypopituitarism and related disorders. The available evidence to date suggests that this defect should be suspected in patients with pituitary hormone deficiencies and craniofacial phenotypes, even in the absence of retinal dystrophy, oral phenotype, intellectual disability, or postaxial polydactyly. Finally, our results generated a developmental human brain expression profile of *TBC1D32*, confirmed the role of TBC1D32 as an interacting partner of HDAC1, and highlighted its connections to cilia assembly, SHH signal transduction, the immune system, and the regulation of the cell cycle. The immune system and cell cycle–related TBC1D32 functions may modify the phenotype of patients with *TBC1D32* mutations, and suggest an interesting direction for future studies.

## Abbreviations

ACTH: adrenocorticotropin
AP: affinity purification
CCRK: cell-cycle related kinase
cDNA: complementary DNA
CNS: central nervous system
CS: Carnegie stage
CTG-scan: cardiotocography
ECG: electrocardiogram
GAP: GTPase-activating protein
GH: growth hormone
GW: gestational week
HCIP: high confidence interacting protein
HDAC1: histone deacetylase I
IGF-1: insulin-like growth factor 1
IGFBP3: insulin-like growth fact or-binding protein 3
MRI: magnetic resonance imaging
MS: mass spectrometry
OFDS: oral-facial-digital syndrome
PCR: polymerase chain reaction
PDA: patent ductus arteriosus
SDS: standard deviation score
Shh/SHH: Sonic Hedgehog
TSH: thyroid-stimulating hormone
WGS: whole genome sequencing

## Additional Information

***Disclosure summary*:** The authors have nothing to disclose.

***Data Availability:*** All data generated or analyzed during this study are included in this published article or in the data repositories listed in References.
